# An AGS-associated mutation in ADAR1 catalytic domain results in early-onset and MDA5-dependent encephalopathy with IFN pathway activation in the brain

**DOI:** 10.1186/s12974-022-02646-0

**Published:** 2022-12-01

**Authors:** Xinfeng Guo, Richard A. Steinman, Yi Sheng, Guodong Cao, Clayton A. Wiley, Qingde Wang

**Affiliations:** 1 Department of Surgery, University of Pittsburgh School of Medicine, 200 Lothrop Street, Pittsburgh, PA 15213 USA; 2 Department of Medicine, University of Pittsburgh School of Medicine, Pittsburgh, PA USA; 3 Department of Pharmacology and Chemical Biology, University of Pittsburgh School of Medicine, Pittsburgh, PA USA; 4 Magee Women Research Institute, University of Pittsburgh, Pittsburgh, PA USA; 5 Department of Neurology, University of Pittsburgh School of Medicine, Pittsburgh, PA USA; 6 Department of Pathology, School of Medicine, University of Pittsburgh, Scaife Hall, 200 Lothrop Street, Pittsburgh, PA 15213 USA; 7 V.A. Pittsburgh Health System, Pittsburgh, PA USA; 8 Pittsburgh Liver Research Center, University of Pittsburgh Medical Center and University of Pittsburgh School of Medicine, Pittsburgh, PA USA

**Keywords:** Aicardi–Goutières syndrome (AGS), Neurodegeneration, Neuroinflammation, Innate immunity, ADAR1, Gene mutation, Mouse model, MDA-5, RNA sensing signaling pathway

## Abstract

**Background:**

Aicardi–Goutières syndrome (AGS) is a severe neurodegenerative disease with clinical features of early-onset encephalopathy and progressive loss of intellectual abilities and motor control. Gene mutations in seven protein-coding genes have been found to be associated with AGS. However, the causative role of these mutations in the early-onset neuropathogenesis has not been demonstrated in animal models, and the mechanism of neurodegeneration of AGS remains ambiguous.

**Methods:**

Via CRISPR/Cas-9 technology, we established a mutant mouse model in which a genetic mutation found in AGS patients at the ADAR1 coding gene (*Adar*) loci was introduced into the mouse genome. A mouse model carrying double gene mutations encoding ADAR1 and MDA-5 was prepared using a breeding strategy. Phenotype, gene expression, RNA sequencing, innate immune pathway activation, and pathologic studies including RNA in situ hybridization (ISH) and immunohistochemistry were used for characterization of the mouse models to determine potential disease mechanisms.

**Results:**

We established a mouse model bearing a mutation in the catalytic domain of ADAR1, the D1113H mutation found in AGS patients. With this mouse model, we demonstrated a causative role of this mutation for the early-onset brain injuries in AGS and determined the signaling pathway underlying the neuropathogenesis. First, this mutation altered the RNA editing profile in neural transcripts and led to robust IFN-stimulated gene (ISG) expression in the brain. By ISH, the brains of mutant mice showed an unusual, multifocal increased expression of ISGs that was cell-type dependent. Early-onset astrocytosis and microgliosis and later stage calcification in the deep white matter areas were observed in the mutant mice. Brain ISG activation and neuroglial reaction were completely prevented in the *Adar D1113H* mutant mice by blocking RNA sensing through deletion of the cytosolic RNA receptor MDA-5.

**Conclusions:**

The *Adar D1113H* mutation in the ADAR1 catalytic domain results in early-onset and MDA5-dependent encephalopathy with IFN pathway activation in the mouse brain.

**Supplementary Information:**

The online version contains supplementary material available at 10.1186/s12974-022-02646-0.

## Background

Mutations in the gene coding the RNA editing enzyme ADAR1 [1, 2], together with six other genes [3–5], have been linked to Aicardi–Goutières syndrome (AGS), an infant or juvenile-onset neurodegenerative disease often leading to severe brain injury manifested by progressive cognitive and intellectual regression, spasticity, dystonia, and motor disability [3, 6, 7]. The outcome of this disease is poor, with most patients not surviving into adulthood [5, 8–10]. The mechanism of AGS has not been completely elucidated and there is no effective therapy to alter the disease course [5, 9]. The notable biochemical feature of AGS is a type 1 IFN “signature” of elevated expression of interferon-stimulated genes (ISGs) detected in the cerebral spinal fluid and blood [7, 8, 11]. Interferon signaling pathway activation is believed to play a central role in the brain pathogenesis [11, 12]. All of the AGS-associated genes encode proteins involved in nucleotide metabolism and/or signaling of DNA/RNA sensing [4, 7, 11], and activation of the nucleic acid sensing pathway leads to activation of the type I interferon signaling pathway, the innate immune pathway well-defined in the defense against viral infections [13–15]. It has been assumed that AGS is caused by accumulation of aberrantly processed DNA/RNA, which is detected by the cellular DNA/RNA sensing signaling pathway, resulting in inflammatory ISG cytokine production and neurodegeneration [3, 4, 11].

Mutant animal models have been prepared to study the pathogenesis of AGS [12]. Activation of the IFN-signaling pathway has been observed in mice carrying knockout or mutant AGS genes [2, 4, 16–19], supporting a causative link between the mutations and interferon signaling pathway activation. However, despite the importance of these models to define the disease mechanisms, they failed to recapitulate the disease phenotypes observed in humans. Of particular note, the phenotype of the predominant brain pathogenesis of AGS has not been replicated in any murine system [12]. There is a gap in our understanding between genetic mutations and brain injury, such as how ISG expression is regulated in the brain and the association between ISG expression and brain injuries in AGS patients. Recently, mouse models of ADAR1 gene mutations were reported in which ISGs were expressed in multiple organs, including the brain [2, 20–24]. Although these models connected ADAR1 mutations with ISG expression, they did not recapitulate the early-onset brain-dominant pathological features of AGS.

In this study, we report a novel mouse model that better resembles the genetic and phenotypic features of AGS. It carries a mutation in ADAR1 catalytic domain, which is equivalent to the *Adar D1113H* mutation found in AGS patients. The phenotype of brain-dominant inflammatory injury developed in *Adar D1113H* mutant mice. The significantly elevated ISG levels were observed in the brains of *Adar D1113H* mice as early as birth and were dramatically increased in 2-week-old mice and caused mild gliotic responses by 8 weeks of age. At late stages, mice bearing this mutation exhibited calcium deposition in the deep gray matter (DGM) regions. We assessed ISG expression in situ and characterized the spatial and cell-type specific patterns of ISG expression. Finally, we found that the brain ISG activation and gliosis were completely prevented in the *Adar D1113H* mutant mice by blocking RNA sensing through deletion of the cytosolic RNA receptor MDA-5, indicating that MDA-5 was central to ISG expression in the brain.

## Results

### *Adar D1113H* mutation results in growth retardation in postnatal mice

**Fig. 1:**
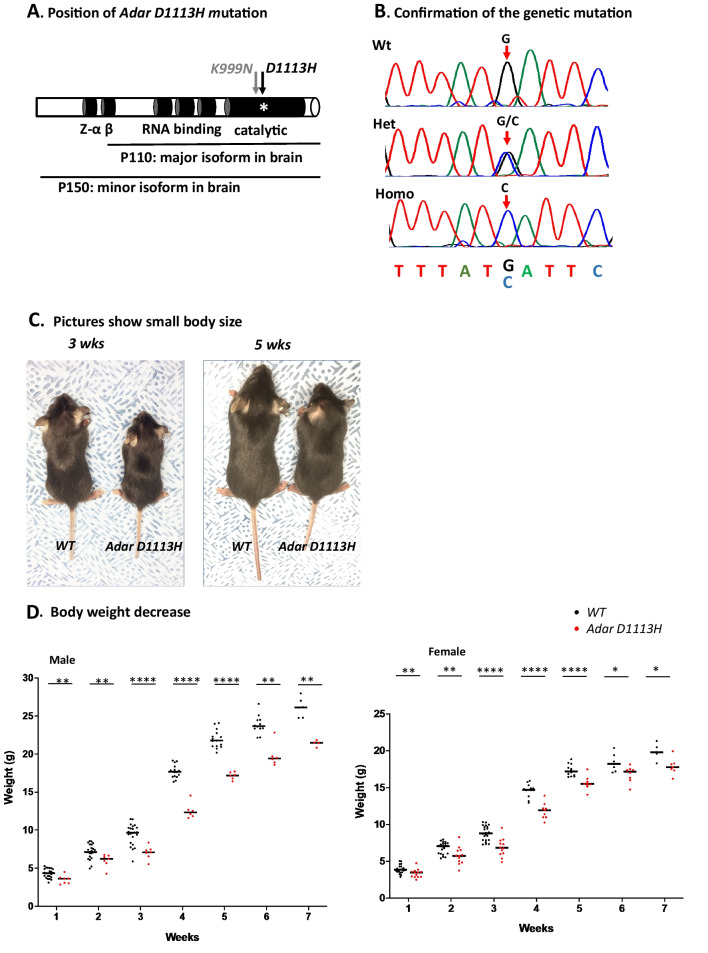
*Adar D113H* mutation results in growth retardation in mice. **A** Using CRISPR/Cas9 technology, a single nucleotide guanosine (G) to cytidine (C) mutation was introduced into the mouse genome that codes the *Adar D963H* mutation in mice, equivalent to the *Adar D1113H* mutation found in AGS patients. The mutation sites of *D1113H* and the adjacent mutation *K999N* in the catalytic domain are indicated with arrows. The protein structure of ADAR1 and the functional domains and isoforms of P150 and P110 are indicated. **B** The genome G > C replacement was confirmed by Sanger sequencing in the *Adar D1113H* mouse strain founder and the progenies. The G and C peaks in the Sanger sequencing chromatograms are indicated by arrows*.* **C** The homozygous *Adar D1113H* mice showed a smaller body size, as shown at three and 5 weeks of age. **D** and **E** The body weight of the male and female mice was significantly lower in *Adar D1113H* mice than in the littermate controls from one to 7 weeks of age. Data were analyzed using Mann–Whitney *U*-test at each timepoint, *n* = 4–23 (male), 5–22 (female). *p < 0.05; **p < 0.01; *p* < 0.001, ****p < 0.0001. Lines indicate medians

Using CRISPR/Cas9 techniques, we successfully introduced a G > C nucleotide replacement into the mouse genome to encode the D963H mutation of ADAR1 in the catalytic domain, equivalent to the *Adar D1113H* mutation found in AGS patients [1, 10], Fig. 1A and B.

For convenient cross-reference with the human AGS mutation, we refer below to this specific mutation as *Adar D1113H* mutation, and this mouse strain as *Adar D1113H* mutant mice. The mutation was confirmed in the founder and in progenies by Sanger sequencing, Fig. 1B. The colony was expanded for analysis and the mice were genotyped using optimized PCR conditions to distinguish the mutant allele from the wild type (WT), Additional file 1: Fig S1. We selected homozygous mice for pathologic analysis, since only homozygous *Adar D1113H* mutations have been found in AGS patients [1, 10] and because no gross phenotype was observed in heterozygous mice.

ADAR1 mutation in AGS patients results in severe brain injuries beginning at birth and is manifested by irritability, dystonia, microcephaly, intellectual disability, spastic dystonia, and deficient gross motor function [1, 10]. Patients die in their teens with phenotypic neurodegeneration. In contrast to patients, homozygous mice do not show abnormalities at birth or in the first 3 weeks before weaning. A growth retardation phenotype develops from one week of age without an apparent feeding problem and body size and weight consistently remain low through adulthood, Fig. 1C–E. However, these mice did not exhibit significant health problems and grew to adulthood without excess mortality and no evidence of spasticity or dystonia up to 13 months. Both male and female mice were fertile with normal litter size.

## *Adar D1113H* mutation results in prominent ISG expression in the brain

**Fig. 2:**
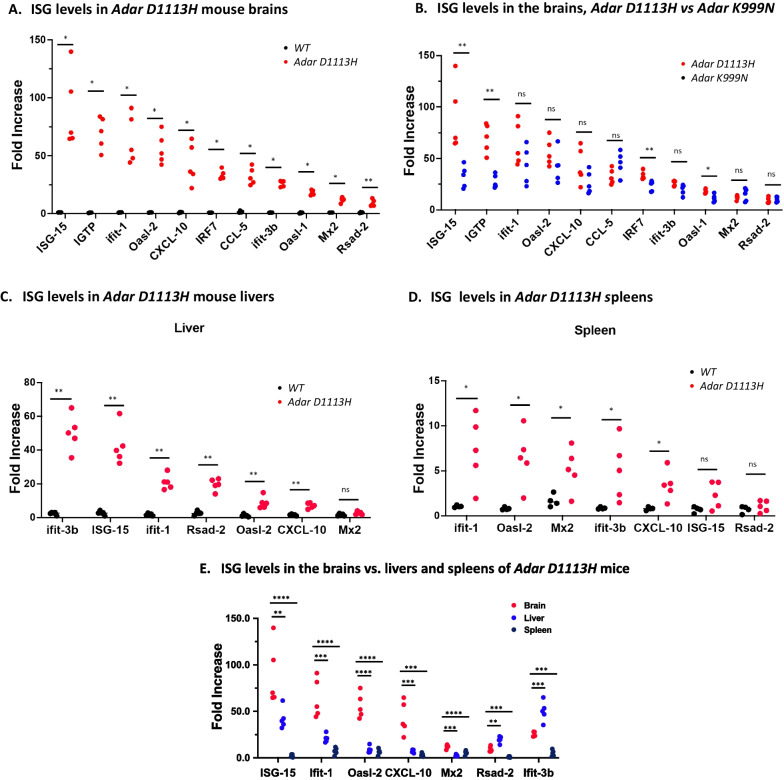
ISG expression levels were elevated in *Adar D1113H* mice. **A** Interferon-stimulated gene (ISG) expression in brain tissues of WT and *Adar D1113H* mutant mice was quantified using a selected real-time PCR panel covering 11 ISGs as indicated. Within this panel, mRNA levels of all ISGs were significantly increased in *Adar D1113H* mice, with more than 50-fold increases of ISG-15, IGTP, Ifit-1, and Oasl-2 genes. **B** ISG expression levels in the brains of *Adar D1113H* mice were compared with those in the brains of *Adar K999N* mice. ISG-15, IGTP, IRF7, and Oasl-1 levels were significantly higher in *Adar D1113H* mice than in *Adar K999N* mice. **C** and **D** ISG expression levels in the livers and spleens of *Adar D1113H* mice were assessed. In the livers, expression levels of six of seven tested ISGs were higher in *Adar D1113H* mice than in the controls. Iifit-3b and ISG-15 mRNAs increased by more than 40-fold. In the spleen, expressions of five of seven tested ISGs increased by 3- to 6-fold. **E** In *Adar D1113H* mice, ISG expressions in the brain were compared to those in the liver and spleen. All seven tested ISGs were expressed in the brains at a significantly higher level than in the spleens, and five of seven tested ISGs were higher than in the livers. Only Rsad-2 and Ifit-3b were expressed at higher levels in the liver than in the brains. The gene expression levels were calculated using ΔΔt method with reference to the average of GAPDH and HRTP. Increases were expressed as fold changes relevant to levels in wild type mice. Pairwise comparisons (**A**–**D**) were analyzed using Mann–Whitney *U* test (**A**–**D**) and three-way comparison by one-way ANOVA with Tukey adjustment for multiple comparisons. *n* = 5 (wild type and *Adar D1113H* group), *P < 0.05, **P < 0.01, ***P < 0.001, ****P < 0.0001

We assessed whether the *Adar D1113H* mutation was associated with IFN pathway activation in the brain. Total RNA of the whole brain was isolated from mutant and littermate controls at 8 weeks of age. The expression level of a panel of 11 ISGs was assessed using a quantitative real-time PCR approach [2]. ISG levels in *Adar D1113H* mice were dramatically increased compared to littermate controls, Fig. 2A. Among this ISG panel, ISG15, IGTP, Ifit-1, and Oasl-2 RNA levels increased more than 50-fold. Other ISGs (CXCL-10, IRF7, CCL-5, Ifit 3b, MX2, Oasl1, and Rsad 2) also increased their expression levels by 10–40 fold.

We previously found that *Adar K999N* mutation mediated elevated brain ISG expression [2]. To test whether the ISG expression pattern is similar in these two models, we compared the ISG levels in these mutant strains and found that ISG-15, IPTG, IRF7, and Oasl-1 expression was significantly higher in *Adar D1113H* than in *Adar K999N* mice, whereas Ifit-1, Oasl-2, CXCL-10, CCL-5, and Ifit-3b showed similar expression levels between these two mutations, Fig. 2B. In general, the *Adar D1113H* mutation exerted a stronger effect on brain ISG expression.

Dramatically increased ISG expression was also observed in the liver, Fig. 2C. A statistically significant but quantitatively smaller increase was observed in the spleen, Fig. 2D. Increased ISGs in the liver included Ifit-3b, ISG-15, Ifit-1, Rsad-2, and CXCL10. In the spleen, Ifit-1, Oasl-2, Mx2, Ifit-3b, and CXCL-10 were increased. However, the ISG levels in the liver and spleen were not as high as those in the brains. The levels of ISG-15, Ifit-1, Oasl-2, CXCL10, and MX2 were significantly higher in the brain, while only Rsad-2 and Ifit-3b showed higher levels in the liver than in the brain, Fig. 2E.

## ISG expression begins around birth and reaches adult levels by 2 weeks in *Adar D1113H* mutant mice

**Fig. 3:**
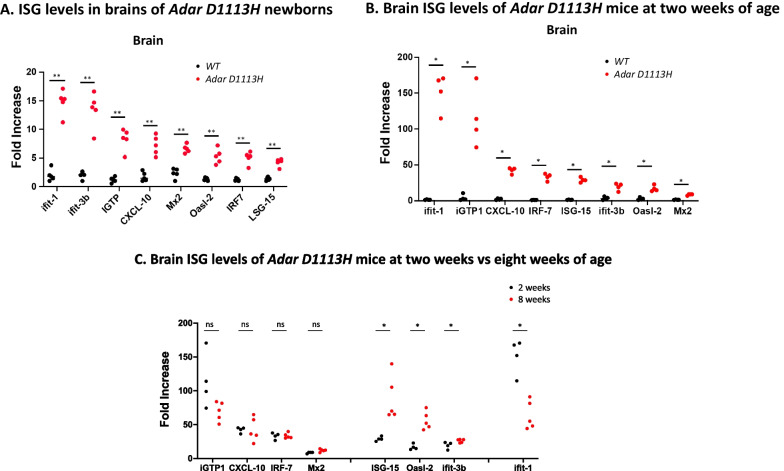
Age-dependence of brain ISG expression in *Adar D1113H* mutant mice. **A** Newborn mice exhibited heightened expression of ISGs compared with WT mouse brains. **B** Expression at 2 weeks remained elevated, increasing for some ISGs. **C** Comparison of ISG expression in brains at 2 versus 8 weeks. Pairwise comparisons between genotypes or ages for each ISG was performed using Mann–Whitney *U* test (*n* = 4–6 per group)

To determine why the *Adar D1113H* mutation failed to cause early neurological signs and symptoms that characterize AGS patients, we looked closer at the onset of ISG expression. We compared ISG expression in pre-weaned mice at 2 weeks of age with that of newborns. RNAs were isolated from whole brain tissue and ISG expression levels were measured. As shown in Fig. 3A, B, significant ISG expression was observed in the brains of the newborn and the 2-week-old mutant mice compared to littermate controls. All eight tested ISGs were significantly increased in newborn and 2-week-old mice. This was well demonstrated with Ifit-1 and IGPT in 2-week-old mice, where expression levels showed a more than 100-fold increase. CXCL-10, IRF-7, ISG-15, IFit-3b, and Oasl-2 showed a more moderate increase of 10- to 40-fold, while Mx-2 increased less, Fig. 3B. While ISG levels were higher in newborn mutant mice than in WT mice at birth, they continued to increase in the mutant mice up to tenfold higher than the newborn levels over the next 2 weeks. Further increase during adulthood varied among the ISGs. ISG levels of IGTP, CXCL10, IRF7, and MX2 were comparable between 2- and 8-week-old mice, whereas levels of ISG-15, Oasl2, and Ifit-3b were significantly higher (and Ifit-1 was significantly lower) in the older mice, Fig. 3C. These results show that the IFN-signaling pathway was activated as early as birth, rapidly increased in neonates, and was maintained at a high level through adulthood.

## Different expressions of distinct ISGs in brain cell types

**Fig. 4:**
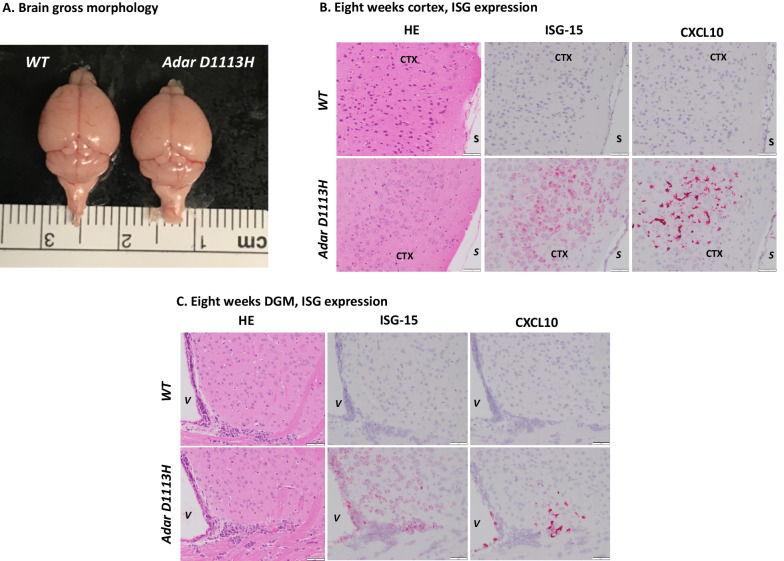
Distribution of ISG expression in *Adar D1113H* mutant mouse brain determined by RNA in situ hybridization (ISH). **A** Brains of WT and *Adar D1113H* mutant mice photographed at 8-week-old are shown. An apparent difference in gross morphology was not observed. **B** and **C** Formalin fixed paraffin embedded (FFPE) sections of 8-week-old mice were stained with hematoxylin and eosin (H&E) (left) or ISH for ISG-15 (middle) or CXCL10 (right). **B** Cortical sections (S = pial surface, CTX = cortex) show no evidence of inflammatory infiltrate on H&E stain in WT or mutant mice. ISH in mutant mice shows strong labeling of cortical neurons for ISG-15 (middle) and microglia for CXCL10 (right) in mutant but not WT mice. **C** Deep gray matter sections (V = lateral ventricle, CP = choroid plexus) show no evidence of inflammatory infiltrate on H&E stain in WT or mutant mice. ISH in mutant mice shows strong labeling of cortical neurons and ependyma for ISG-15 (middle) and microglia and ependyma for CXCL10 (right) in mutant, but not WT mice. Bar = 50 microns

ISGs were highly expressed in the brain from the newborn stage or even before birth, which potentially impacted early brain development; however, the gross morphology of the brain did not show an apparent difference between *Adar D1113H* and WT mice, Fig. 4A. To assess the ISG expression pattern in the brain and characterize pathologic features associated with ISG expression, chromogenic ISH was performed on brain sections with select probes for ISG-15 and CXCL10 as previously described [2]. ISH studies showed abundant expression of ISG15 within central nervous system (CNS) neurons and CXCL10 within microglia, Fig. 4B, C. The staining pattern showed an unusual multifocal characteristic that did not specifically comply with neuroanatomical regions. With the ISG15 probe, strong and diffuse staining was observed in broadly distributed neurons in the cortex and DGM areas. Staining was greater in superficial cortical neurons than in deep neurons. There also was intense staining of ependymal and choroid plexus epithelium. Scattered cells in the deep white matter were also stained. With the CXCL10 probe, a completely different staining pattern was shown by ISH, Fig. 4B, C. Scattered foci of microglia showed intense staining. There was intense staining of ependyma and choroid plexus macrophages. There was no staining of neuronal elements with the CXCL10 probe, Fig. 4.

## *Adar D1113H* mutation was associated with mild astrocytosis and microgliosis in 8-week-old mice, which was exacerbated in aged mice

**Fig. 5:**
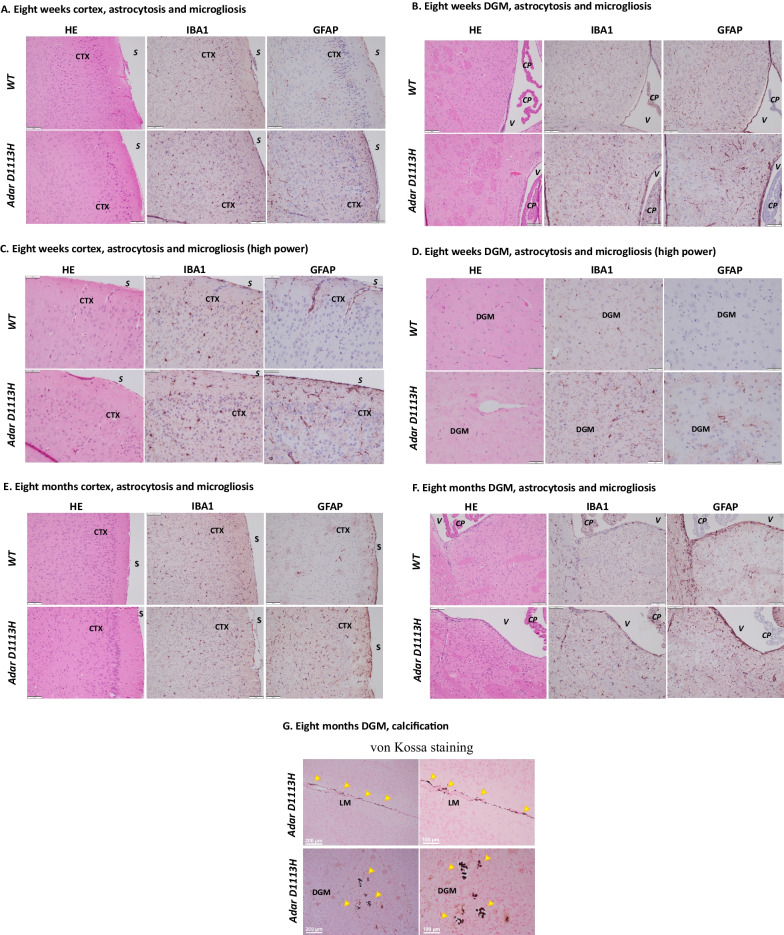
*Adar D1113H* results in astrocytosis and microgliosis in 8-week-old mice. FFPE sections of 8-week-old (**A–D**) and 8-month-old (**E–F**) mice were stained with hematoxylin and eosin (H&E) (left) or immunohistochemistry (IHC) for IBA1 (middle) or GFAP (right). **A** Cortical sections (S = pial surface, CTX = cortex) showed no evidence of inflammatory infiltrate on H&E stain in WT or mutant mice. IHC in mutant mice showed mildly increased labeling of microglia with IBA1 (middle) and astrocytes with GFAP (right) in mutant compared to the WT mice. Bar = 100 microns. **B** Deep gray matter sections (V = lateral ventricle, CP = choroid plexus) showed no evidence of inflammatory infiltrate on H&E stain in WT or mutant mice. IHC in mutant mice showed mildly increased labeling of microglia with IBA1 (middle) and astrocytes with GFAP (right) in mutant compared to the WT mice. Bar = 100 microns. **C** High power image of cortical sections (CTX = cortex). H&E-stained sections of WT and *Adar D1113H* brains showed normal distribution of cortical neurons with interspersed blood vessels. Leukocyte infiltration or perivascular cuffs were not observed. IHC staining for IBA1 showed comparable dark staining of cell bodies in both WT and *Adar D1113H* brains. There was a slightly subjectively greater prominence of delicate microglial processes, but this finding was mild. Similarly, IHC staining for GFAP showed moderate perivascular staining of astrocyte endfoot processes along penetrating blood vessels. More smaller vessels showed GFAP staining in the *Adar D1113H* brains along with mildly greater prominence of astrocytic parenchymal processes. Bar = 50 microns. **D** High power image of deep gray matter (DGM) sections. H&E-stained sections of WT and *Adar D1113H* brains show normal distribution of DGM neurons with interspersed blood vessels. Leukocyte infiltration or perivascular cuffs were not observed. In WT mice (top) IHC staining for IBA1 showed occasional dark staining of cell bodies with delicate cell processes. IBA1 staining in *Adar D1113H* brains (bottom) showed more cell bodies and a coarsening and darker staining of microglial processes. There was a slightly subjectively greater prominence of delicate microglial processes, but this finding was mild. Similarly, IHC staining for GFAP in WT mice showed only faint perivascular staining of astrocyte endfoot processes along penetrating blood vessels. More smaller vessels showed GFAP staining in the *Adar D1113H* brains along with greater prominence of astrocytic parenchymal processes. Bar = 50 microns. **E** Cortical sections (S = pial surface, CTX = cortex) showed no evidence of inflammatory infiltrate on H&E stain in WT or *Adar D1113H* mutant mice. IHC in mutant mice showed mild increased labeling of microglia with IBA1 (middle) and astrocytes with GFAP (right) in *Adar D1113H* mutant compared to the WT mice. Bar = 100 microns. **F** DGM sections (V = lateral ventricle, CP = choroid plexus) showed no evidence of inflammatory infiltrate on H&E stain in WT or *Adar D1113H* mutant mice. IHC in mutant mice showed mildly increased labeling of microglia with IBA1 (middle) and astrocytes with GFAP (right) in *Adar D1113H* mutant compared to WT mice. Bar = 100 microns. **G** FFPE sections of 8-month-old mutant mice stained using von Kossa technique for calcium (brown/black). Perivascular calcification is noted in the leptomeninges (LM) and DGM areas. Bar = 200 microns (left) and 100 microns (right)

We assessed whether ISG expression in *Adar D1113H* mutant mice results in brain pathologic changes. The *Adar D1113H* mutant mouse brains were histologically evaluated at 8 weeks of age. Despite the highly expressed ISGs in the brain cells, no apparent neuron loss or inflammatory cell infiltration was noted in the brains of 8-week-old mice. Hematoxylin and eosin (H&E)-stained brain sections revealed unremarkable neuroglial parenchyma, Fig. 5A, B. To more sensitively assess subtle neuropathological changes, brains were immunohistochemically stained for glial fibrillary acidic protein (GFAP) and ionized calcium-binding adapter molecule 1 (IBA1) to discern astrocytosis or microgliosis, respectively. Mild microgliosis and astrocytosis was observed in the cortex, Fig. 5A, and DGM areas, Fig. 5B.

As observed under higher power microscopy, H&E-stained sections of *WT* and *Adar D1113H* brains show normal distribution of cortical neurons, Fig. 5C, and DGM neurons, Fig. 5D, with interspersed blood vessels. Evident leukocyte infiltration or perivascular cuffs were not observed. In cortex areas, IBA1 staining showed comparable dark staining of cell bodies in both *WT* and *Adar D1113H* brains. There was a slightly subjectively greater prominence of delicate microglial processes, Fig. 5C. Similarly, GFAP staining shows moderate perivascular staining of astrocyte endfoot processes along penetrating blood vessels. More smaller vessels showed GFAP staining in the *Adar D1113H* brains along with a mildly greater prominence of astrocytic parenchymal processes, Fig. 5C. In DGM areas, IBA1 staining showed occasional dark staining of cell bodies with delicate cell processes in *WT* mice, while it showed more cell bodies and coarsening and darker staining of microglial processes in *Adar D1113H* brains, Fig. 5D. GFAP staining in WT mice showed only faint perivascular staining of astrocyte endfoot processes along penetrating blood vessels. More smaller vessels showed GFAP staining in the *Adar D1113H* brains along with greater prominence of astrocytic parenchymal processes, Fig. 5D.

We also assessed the pathologic changes in mutant mice at 8 months of age. There were no noticeable changes observed in H&E-stained brain sections, Fig. 5E, F. ISG expression was confirmed by RNA ISH, and the staining pattern was similar to that of the 8-week-old mice, Additional file 1: Fig S3, S4. However, the IBA-1 and GFAP staining showed an increased microglial and astrocytic reaction in the *Adar D1113H* mutant mice compared to WT mice, Fig. 5E, F in both the cortex and DGM areas. Von Kossa staining showed microscopic foci of vascular calcification in DGM regions, Fig. 5G.

## *Adar D1113H* mutation alters RNA editing activities in the brain

ADAR1 catalyzes A-to-I RNA editing through its deamination activity, which converts adenosine to inosine and modifies sequences of RNA substrates. The *Adar D1113H* mutation lies in the catalytic domain of ADAR1, where seven of the nine originally reported AGS-associated *Adar* mutations reside [1]. Although *Adar D1113H* does not lie in the active binding site or RNA interface of ADAR1, structural mapping of human ADAR2-dsRNA complexes indicates that the *Adar D1113H* mutation likely causes conformational changes that interfere with RNA binding [25]. An earlier in vitro study showed that the *Adar D1113H* mutation decreased ADAR1’s editing activity on an RNA substrate [1]. To determine a potential relationship between ISG expression and a deficit in RNA editing in *Adar D1113H* mutant mice, we assessed the editing activity in the mutant mice. First, we confirmed that a normal sized ADAR1 protein was expressed from the *Adar D1113H* mutant gene in the brain, Additional file 1: Fig S2.

**Fig. 6:**
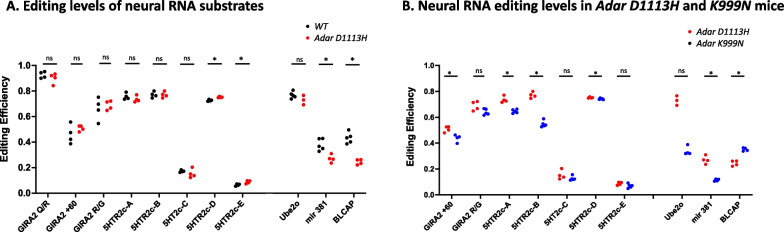
Effect of *Adar D1113H* mutantion on RNA editing. Neural RNA transcripts in *Adar D1113H* mutant mice were assessed for their RNA editing levels at the known editing sites in defined A-to-I RNA editing substrates, including editing sties in neuron receptors GIRA2 and 5-HT2cR, as well as the editing sites in mRNAs for BLCAP, Ube2o mRNAs, and microRNA 381. **A** RNA editing levels in *Adar D1113H* mouse brains were compared with that in the wildtype control mice. Editing activity of *Adar D1113H* mutation was significantly decreased at the editing sites in microRNA 381 and BLCAP mRNAs, whereas the editing sites in neuron receptor mRNAs and Ube2o mRNAs did not show a difference or only minimal differences. **B** Neural RNA editing level in *Adar D1113H* mutant mice was compared with that of *Adar K999N* mutant mice. These two mutant mice showed different RNA editing patterns in the tested neural RNA transcripts. The editing at the GIRA2 intron + 60 site, 5-HTR2c A, B, and D sites, and in miroRNA381 was significantly lower in *Adar D1113H* mice than in *Adar K999N* mice, whereas BLCAP mRNA editing was higher in *Adar D1113H* mice. Pairwise comparison of editing of each site between these two mutant groups was conducted using Mann–Whitney *U* test (*n* = 4–5 mice per group). *p < 0.05

Next, we assessed whether the *Adar D1113H* mutation impacted editing activity in the brain by analyzing editing levels on brain transcripts known to be edited by ADAR1, including the GRIA2 + 60 intron site, GIRA2 R/G site, and 5-hydroxytryptamine receptors 2c (5-HTR2c) sites (A, B, C, and E sites). As a negative control, we also measured editing at the GIRA2 Q/R sites and 5-HTR2c D site that are targeted by ADAR2 rather than by ADAR1 [17, 26]. As expected, editing at the GIRA2 Q/R sites was not affected in *Adar D1113H* mutant mouse brains and the 5-HTR2c-D site only minimally differed between *WT* and *Adar D1113H* mice (0.74 versus 0.75). Interestingly, the *Adar D1113H* mutant mice did not differ from *WT* mutant mice in six of seven tested sites in neuron receptor mRNAs known to be edited by ADAR1, whereas only the 5HTR2c-E site showed a minimal increase, Fig. 6A. We further investigated additional RNA editing substrates known to be edited by ADAR1 in microRNA381 and the mRNAs of BLCAP and Ube2o [27]. We found that editing in microRNA381 and BLCAP mRNA were significantly decreased by the *Adar D1113H* mutation, while there was no significant difference in editing of Ube2o mRNA, Fig. 6A.

We previously reported on mice bearing another AGS-associated mutant in the catalytic domain of *Adar K999N* [2]. Structure-based analysis of ADAR1 suggested that both the K999N site and the D1113H site are situated at positions that determine RNA substrate binding [25], so that a similar effect on editing activity could be expected between these mutations. However, the results showed that these two mutants led to different editing patterns. While both of the mutant mice exhibited the same editing activity on the GIRA2 R/G site, 5-HT2c-C, E sites, and on Ube2o mRNAs, there were significant differences in the editing levels on the sites of GRIA2 + 60 and 5-HT2c A, B, and D sites, as well as in the editing levels on microRNA381 and BLCAP mRNA. While the *Adar K999N* mutation had lower levels of editing on the 5-HTR2c A, B, and D and miR381 editing sites than *Adar D1113H* mutation, the editing level on BLCAP was higher in mice bearing the *Adar K999N* mutation, Fig. 6B. In general, the *Adar D1113H* mutation had a lesser effect on the tested RNA substrates than *Adar K999N*. We note that even though the *Adar D1113H* mutation edited fewer of these select substrates in the brain than the *Adar K999N* mutation, the *Adar D1113H* mice exhibited a higher level of ISG induction in their brains than *Adar K999N* mice.

## Deletion of MDA-5 prevents ISG expression, astrocytosis, and gliosis in mutant mice

Next, we tested whether the ISG expression in the brains of *Adar D1113H* mice depended upon the cytoplasmic RNA receptor melanoma differentiation-associated protein 5 (MDA-5). MDA5 recognizes double-stranded RNA and triggers interferon pathways following interaction with MAVS (mitochondrial antiviral signaling protein) [13–15]. MDA-5 has been shown to be required for IFN pathway activation in ADAR1-deficient mouse models [20–24, 28, 29] and deletion of MDA-5 completely rescues the lethality in catalytic null Adar1^E861A/E861A^ mutant mice [18, 28]. However, the postnatal lethality of *Adar1*^*∆7–9*^ [19], *Adar1*^*∆2–13*^ [30], *Adar1*^*∆12–15*^ [31], and *P150*^*−/−*^ [19] could not be rescued by either MDA-5 or MAVS deletion. Whether the phenotype observed in ADAR D1113H mutant mice is MDA-5-dependent was yet to be determined.

**Fig. 7:**
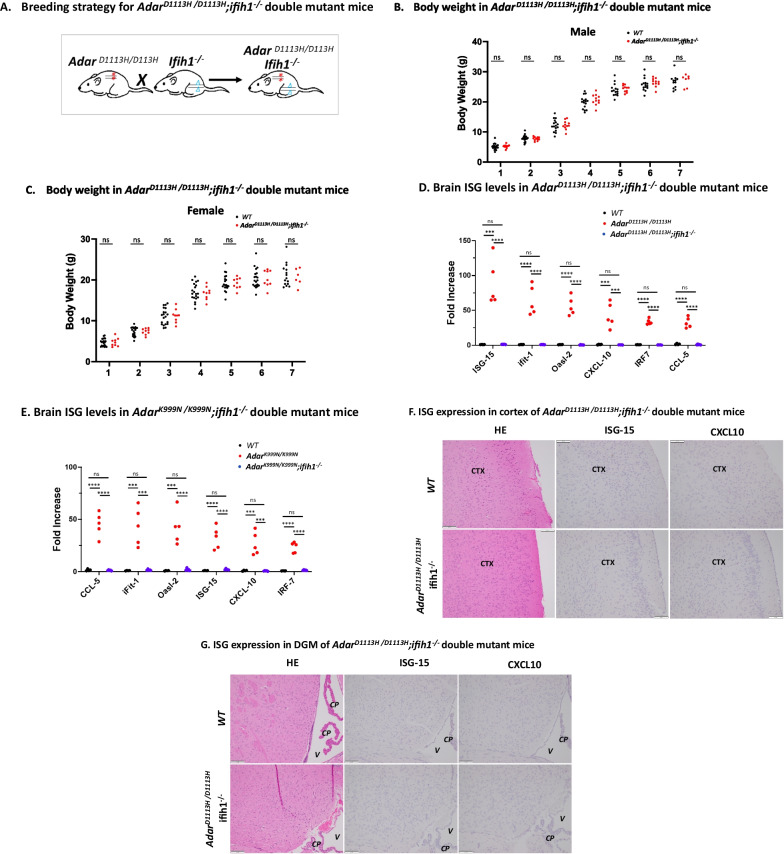
MDA-5 dependence of ADAR1 mutant growth inhibition, ISG expression, and brain pathology. **A** *Adar*^*D1113H /D1113H*^*;ifih1*^*−/−*^ double-mutant mice were generated by crossing the two mutant strains followed by genotype selections. In *Adar*^*D1113H /D1113H*^*;ifih1*^*−/−*^ double-mutant mice, the effect of *Adar*^*D1113H /D1113H*^ mutation was tested in mice without the presence of MDA-5. **B**, **C** Body weight of *Adar*^*D1113H /D1113H*^*;ifih1*^*−/−*^ double-mutant mice were measured separately for male and females from one to 7 weeks of ages. Both male and female mice did not exhibit growth retardation in comparison to WT mice. All comparisons at each age were non-significant as determined by Mann–Whitney *U* test (*n* = 7–20 per group). **D** ISG mRNA levels in *Adar*^*D1113H /D1113H*^*;ifih1*^*−/−*^ double-mutant mice, as well as in *Adar*^*K999N /K999N*^*;ifih1*^*−/−*^ double-mutant mice (**E**), were compared to WT mice. ISG levels in double-mutant mice were significantly lower than in the mutant mice and not different from the wildtype control mice. One-way ANOVA with Tukey adjustment for multiple comparisons, *n* = 4–5 per group; p < 0.001; ****p < 0.0001. **F** Formalin fixed paraffin embedded (FFPE) sections of 8-week-old mice were stained with hematoxylin and eosin (H&E) (left) or in situ hybridization (ISH) for ISG-15 (middle) or CXCL10 (right). Cortical sections (CTX) show no evidence of inflammatory infiltrate on H&E stain in WT or mutant mice. ISH in WT and double-mutant mice showed no labeling of cortical neurons for ISG-15 (middle) and microglia for CXCL10 (right). **G** Deep gray matter sections (V = lateral ventricle, CP = choroid plexus) showed no evidence of inflammatory infiltrate on H&E stain in WT or double-mutant mice. ISH in WT and double-mutant mice showed no labeling of cortical neurons and ependyma for ISG-15 (middle) and microglia and ependyma for CXCL10 (right). Bar = 100 microns

We generated an ADAR1 and MDA-5 double-mutant mouse strain by crossing the *Adar D1113H* mutant and *Ifih-1*^*−/−*^ mice [14], Fig. 7A. The double-mutant mice were healthy and indistinguishable from WT mice. In contrast to *Adar*^*D1113H /D1113H*^ mutant mice that exhibited decreased weight compared to WT mice, there was no significant weight difference between WT mice and the *Adar*^*D1113H /D1113H*^; *Ifih1*^*−/−*^ double mutants, Fig. 7B, C.

We assessed the cytokine levels in RNA samples of double-mutant mouse brains. The ISG expression levels in the *Adar* *Ifit1*^*−/−*^ double-mutant brains exhibited the same level as control mice for all tested ISGs, Fig. 7D. We also crossed *Adar K999N* mutant mice with *Ifih1*^*−/−*^ mice. A similar result was observed in the *Adar*^*K999N/K999N*^; *Ifih1*^*−/−*^ double-mutant mice, Fig. 7E, all tested ISGs were at WT control levels.

We also carried out pathologic studies on the double-mutant mice. The elevated levels of ISG15 and CXCL10 that were present in foci of 8-week-old *Adar D1113H* mouse brains were absent in brains from  *Adar*^*D1113H /D1113H*^; *Ifih1*^*−/−*^ mice stained for these cytokines by RNA ISH. Under this analysis, the double-mutant mouse brains were indistinguishable from the 8-week-old WT mouse brains, Fig. 7F, G. Moreover, staining for IBA1 and for GFAP of the double-mutant mouse brains was indistinguishable from that in WT mice, indicating that loss of MDA-5 could prevent astrocytosis and microgliosis, Additional file 1: Fig S5, S6.

## Discussion

We report a novel AGS mouse model in which a single nucleotide replacement encoding an *Adar D1113H* mutation in the catalytic domain of ADAR1 resulted in early-onset IFN-signaling pathway activation in the brain, leading to mild microgliosis and astrocytosis at 8 weeks of age. In 8-month-old mutant mice, calcification in the DGM was observed in addition to microgliosis and astrocytosis. We demonstrated that blocking the RNA sensing pathway by deleting the cytosolic RNA receptor MDA-5 not only normalized ISG expression, but also prevented brain pathology in ADAR D1113H mice, including astrocytosis and microgliosis. Thus, this study demonstrated that an AGS-associated genetic mutation is a causative factor of the early-onset neuropathogenesis of AGS. The study illuminated the molecular consequence of this mutation in RNA transcript editing, as well as the signaling pathway underlying the disease development.

Mutations in seven protein-coding genes have been found to be associated with AGS. However, mouse models bearing these mutations did not sufficiently capture the clinical and neurological characteristics of AGS [12]. Mouse models carrying ADAR1 mutations have been reported recently [2, 20–24]. The pathology changes observed in these models were more striking in the peripheral tissues than in the brain [20–22, 24]. ISGs were expressed in the mutant brains in some of these models [2, 24, 29], but they did not cause early-onset encephalopathy. Various ADAR1 knockout [17, 32–36] and mutant mouse models, including *E861A* [18, 28], *W197A* [29] and *Y177A* [20, 22], were used for ADAR1 mechanistic studies, whereas these mutations have not yet been identified in AGS patients. AGS-associated ADAR1 mutations such as *Adar P195A* (equivalent to *Adar P193A* in humans) [21] and *K948N* (equivalent to *Adar K999N*) [2, 24] were recently reported in mouse models. In AGS, the *Adar P193A* mutation is present as a compound heterozygous mutation. Mice bearing analogous heterozygous *Adar P195A* and a P150 null allele exhibited ISG upregulation in the brain; however, brain pathology was not reported, whereas homozygous *Adar P195A* did not differ from *WT* in its phenotype [4]. *Adar K999N* mutation caused tissue injury in young mice in the liver, lung, kidney, and spleen, but not in the brain; mild neurologic changes and microscopic calcification were observed only in the brains of aged mice [17]. *Adar D1113H* is a mutation found in AGS patients. In our mouse model, this mutation caused robust ISG expression preferentially in the brain and caused early-onset neuroinflammatory responses with astrocytosis and microgliosis.

Our RNA ISH studies on the mutant mouse brain demonstrate widespread multifocal expression of ISGs that does not obey neuroanatomical or neurophysiological boundaries. In addition, different cell types (e.g., neurons and microglia) in the same region show selective expression of distinct ISG. While ISG-15 was expressed in widespread neurons and ependymal cells, CXCL-10 expression was preferential to microglia. This specific ISG expression pattern suggests a microenvironmental stimulus stochastically initiating the ISG expression, since the same gene mutation is in all the cells. However, the regulatory mechanism is currently unknown.

Despite markedly elevated mRNA levels of ISG expression showing a clear “interferon signature” within the nervous system and that elevated protein levels are usually associated with increased coding mRNA levels as shown in *Adar K999N* mice [2], the gross brain morphology was impacted and there was no evidence of significant inflammatory cell infiltration, neuron loss, or other remarkable morphology changes. This was the case despite markedly elevated CXCL10, which has been associated with leukocyte infiltration in several CNS disorders [37], including when artificially expressed in astrocytes [38]. Rather than inflammatory cell infiltration, we observed a mild neuroglia response, including both astrocytosis and microgliosis in 8-week-old mutant mice. These pathologic changes occurred earlier than those reported at one year of age in the *Adar K948N* mice [24]. We also observed DGM calcification in the *Adar D1113H* mutant mice at 8 months of age.

However, *Adar D1113H* mice did not capitulate all the clinical and pathological features of AGS. Although early-onset and brain-predominant inflammation had developed in the mutant mice, severe brain injury manifested by progressive loss of motor ability, spasticity, dystonia, and obvious basal ganglia calcification was not observed. The reason for the difference is not currently known. Since the genetic features of *Adar D1113H* mutation in this mouse model is the same as that in AGS patients, the phenotypic difference might be due to immune response and environmental differences between mice and humans. The mice used in this study were kept in specific pathogen-free (SPF) conditions, which might have prevented environmental factors from exuberating the phenotype.

In AGS patients, most of the mutations in the ADAR1 gene fall in the catalytic domain, and all the homozygous *Adar* mutations, e.g., the three mutations coding *Adar K999N, W1112F,* and *Adar D1113H*, are only found in the catalytic domain [1, 10]. Although *Adar D1113H* is predicted to be a critical amino acid in the catalytic domain [25], decreased editing levels were only observed in mir 381 and BLCAP coding RNAs among the tested sites of known ADAR1 edited RNA substrates in *Adar D1113H* mutant mice, whereas the editing levels of intron + 60 sites on the receptor GIRAs, A and B sites on 5HTR2c and the editing site in Ube2o mRNAs remained the same as those of the control mice. In contrast, *Adar K999N* mutation showed a significant editing level decrease in more tested editing sites, while ISG expression in *Adar K999N* mutant mice was not as high as in *Adar D1113H* mutant mice. Considering that a large number of noncoding sequences are targets of RNA editing, we cannot exclude that there are some untested editing sites that were significantly affected by *Adar D1113H* mutation. A genome-wide measurement of the editing needs to be carried out to determine whether a specific group of RNAs that lost or gained editing with this mutation resulted in ISG expression. It is possible that editing in some specific RNA transcripts inhibits the RNA sensing signaling pathway. Recently, a study suggested that deficient editing of a small group of RNAs is sufficient to activate the IFN pathway [29]. This *Adar D1113H* mutant mouse model will be a useful tool for identification of the specific RNA molecules involved in AGS development.

Previous studies showed that deletion of MDA-5 completely rescued the lethality of the catalytic null *Adar1*^*E861A/E861A*^ mutant mice [18, 28] and prevented ISG expression in some *Adar* mutant mice [20–24, 29]. However, blocking RNA sensing by deletion of MDA-5 or MAVS was not sufficient to prevent the mortality of Adar knockout mice, including *Adar1*^*∆*7–9^ [19], *Adar1*^*∆2–13*^ knockout [30] or *P150*^*−/−*^ mice in which MAVS deletion delayed perinatal death by a few weeks [19, 30]. The *Adar D1113H* mutant mouse is a novel model with profound ISG expression in the brain with early-onset neuropathologic changes. We demonstrated that ISG expression in *Adar D1113H* and *Adar K999N* mutant mouse models depended on MDA-5. Deletion of the MDA-5-encoding *Ifih1* gene normalized ISGs in *Adar D1113H* mice and confirmed the restoration of ISGs in *Adar K999N* mice as previously reported in the *Adar K948N* (equivalent to *Adar K999N*) mouse model [24].

Our study provides evidence that MDA-5 deletion not only normalized ISG expression but also prevented brain pathology in *Adar D1113H* mice, including astrocytosis and microgliosis. The mechanisms linking these pathologic changes to MDA-5-mediated ISG expression are important to elucidate because they could provide a roadmap for treatment in AGS.

## Conclusions

This study demonstrated that the AGS-associated genetic mutation encoding the *Adar D1113H* replacement in the ADAR1 catalytic domain resulted in early-onset neuropathogenesis and illuminated the molecular consequence of this mutation in RNA transcript editing and the signaling pathway underlying the disease development. We demonstrated that the neuroinflammatory pathway activation and brain pathologic changes caused by *Adar* mutations were MDA-5 dependent, which could provide a druggable target for AGS treatment.

## Materials and methods

### Mouse genome mutagenesis

Studies involving the use of mice were approved by the Institutional Animal Care and Use Committee (IACUC) at the University of Pittsburgh. Mouse genome mutagenesis was carried out through the CRISPR/Cas9 gene editing approach in one-cell-stage embryos, as described previously [2]. The donor zygotes were from females with a mixed background of C57 BL/6 J and DBA/2 strains, and we keep the mice in this background for our studies. The potential founders carrying the G > C nucleotide replacement (equivalent to c.3337 g > c in patients) which codes the *Adar D963H* mutation (the *Adar D1113H* mutation in human [1, 10]) were screened among the living pups. Sanger sequencing was used for genome sequence confirmation after PCR amplification of the targeted gene regions. The sgRNA sequence was 5′-ggtctttccggactgtcttt-3′. The ssODN sequence for the G to T mutation at 2997 of ADAR1 gene was 5′-tgtaatacgactcactataggggtctttccggactgtctttgttttagagctagaaat-3′. The primer sequences used for amplifying the region flanking the mutation were 5′-aagtcttgcatgcctgctct-3′ and 5′-ggcacgtgttattcatctgg-3′.

Founders carrying the c.3337 g > c mutations were bred to homozygosity for phenotypic analysis.

### Genotyping analysis

A PCR genotyping approach was established to monitor the genetic status of the progenies. The sequences of the primers were 5′- gccgagtcagtgtttatg-3′ and 5′- gtctggggaaacaaaggcac-3′ for the WT allele and 5′- gccgagtcagtgtttatc-3′ and 5′- catagtcacgggcagctttc-3′ for the mutant allele. The PCR conditions were 94 °C four min., 94 °C 30 s., 63 °C 30 s., and 72 °C 30 s. for 30 cycles. These PCR conditions were optimized to identify single nucleotide replacements and distinguish mutations from the WT gene alleles. After separate PCR reactions for the WT and mutant alleles, the PCR products were combined to run the Agarose gel. The 480-bp band was from the WT allele and the 600-bp band was from the mutant.

### Mouse breeding and phenotype observation

Mice were hosted in a SPF animal facility in the University of Pittsburgh School of Medicine with strict monitoring of temperature, humidity, light cycles, and potential presence of pathogens. Studies were approved by the IACUC at the University of Pittsburgh. The mice were observed from birth to adulthood for growth, behavioral changes, and signs of encephalopathy. The body weights of the mice, together with their littermates, were measured weekly. PCR genotyping was performed on each of the mice at 2 to 3 weeks of age to determine their genetic status. MDA-5 knockout mice were purchased from The Jackson Laboratory, Stock No: 015812. Genotyping was carried out following the Jax protocol for this strain.

### Cytokine expression assays

RNA isolation was performed with RNeasy Plus Mini Kit (Qiagen Cat # 74134) according to the manufacturer’s instructions. Quantitative RT-PCR was performed using the iTaq™ Universal SYBR Green One-Step Kit (Bio-Rad cat #1725151). Assayed genes comprised ISG15, Ccl-5, Ccl-10, Ifit-1, Ifit3, Oasl-1, Oasl-2, Mx2, IL-6, TNF α, Xaf1, IFI 27, Oas1c, IL-1, IFN-α, IFN-β, GAPDH, and HPRT. Primer sequences are listed in the supplemental material. The specificity of PCR amplifications was confirmed by the melting curve and by electrophoresis analysis of the final PCR products. The quantification of the mRNA levels was calculated by the Ct values using ΔΔt method with internal references of the average value of the HPRT and GADH expressions.

### RNA editing essays

Total brain RNA was isolated from undissected whole brains, and reverse transcript PCR was performed with the total RNA samples. The PCR products of the entire brain RNA pool were subjected to Sanger sequencing analysis. The relative quantities of inosine (read as guanosine) and adenosine at each editing site were determined on the chromatographs by the ratio of the G and A peaks. The primer sequences used for the PCR amplifications of the editing sites were 5′-cactgaggaatttgaagatgga-3′ and 5′-agcaggcatggaatgatagg-3′ (for the GRIA2 Q/R site and the intron hot spots), 5′-cttgcgacaccatgaaagtg-3′ and 5′-gccagaaatgtgggtaaagg-3′ (for the GRIA2 R/G site), 5′-agcagagaaagccgtgtgat-3′ and 5′-agaacaccacatccatgcaa-3′ (for the GRIA3 R/G site), 5′-acccgtgcaaccctgact-3′ and 5′-ttgcaggaaattttgtccagt-3′ (for the GRIK1 Q/R site), and 5′-attatgtctggcctttacctagatat-3′ and 5′-ataggaactgaaactcctattgatattgc-3′ (for the editing sites A to E in 5-HT 2cR mRNA). RNA editing efficiency was assessed on the editing sites in GRIA2, GRIA3, GRIK1 mRNAs for the Q/R and R/G sites and on the A–E sites in 5-HT2C receptor mRNA by calculation of the relative ratio of the average of the G peak to the A peak on each of the Sanger sequencing chromatographs for samples from mutant and WT groups.

### Protein sample preparation and analysis

Before tissues were collected from the mice, whole body perfusion was performed with phosphate buffered saline (PBS) with heparin to remove blood from the organs. The tissues were immediately frozen in liquid nitrogen until analysis. For the brain sample collections, the cerebellum and olfactory bulb were removed prior to freezing. Homogenization was performed in RIPA buffer with the addition of protease inhibitor cocktail. ADAR1 protein in the brain tissues was detected using western blot as described previously [18]. In brief, 30 ug of protein extract was loaded to each lane and separated on 8% polyacrylamide gel with 0.1% SDS. ADAR1 was detected with ADAR1 antibody clone 15.8.6 (Santa Cruz sc-73408) at 1:1,000 dilution.

### Pathology studies

Histopathologic studies were carried out on formalin fixed paraffin embedded (FFPE) mouse tissues including the brain, heart, lung, liver, spleen, and kidney. Tissues were harvested and immersion fixed in 4% paraformaldehyde in PBS followed by dehydration, paraffin embedding, and routine pathologic processing with H&E staining.

### Immunohistochemistry

Five-micron-thick sections from paraffin-embedded brain blocks were immunohistochemically stained for GFAP and IBA1 with antibodies; mouse anti-GFAP (catalog# 837202, BioLegend) and rabbit anti-IBA-1 (catalog# WDG5619, WAKO) each at a dilution of 1:1000, followed by secondary antibodies and peroxidase development [39].

### RNA in situ hybridization

ISH studies were performed on FFPE tissue sections using two commercial RNAscope Target Probes (Advanced Cell Diagnostics, Hayward, CA) catalog #559271 and 408921 complementary to sequences 2–561 of ISG-15 and 11–1012 of CXCL10, respectively. Pretreatment, hybridization, and detection techniques (RNAscope 2.5HD) were performed according to manufacturer’s protocols and as previously described [40].

### Statistical analysis

The non-parametric two-sided Mann–Whitney *U* test was used to assess pairwise differences between data, including weight comparisons at specific ages, levels of ISGs between conditions, and RNA editing of specific transcripts compared between two groups of mice. For comparison of ISGs between the brain, liver, and spleen and between WT, mutant, and double-mutant samples, one-way ANOVA was used followed by Tukey adjustment for multiple comparisons. Significance was set at *p* < 0.05 for all comparisons. GraphPad PRISM (version 9.0; GraphPad Software, USA) was used for data analysis.

## Supplementary Information

**Additional file 1: Figure S1.** PCR genotyping analysis of *Adar D1113H* mutant mice. The PCR genotyping condition was optimized to distinguish WT alleles from mutant alleles. Primers used for this genotyping methods yielded two bands of different sizes as indicated. **Figure S2.** *Adar D1113H* mutant mice expressed normal sized ADAR1 protein in brains. Brain protein extracts were prepared from *Adar D1113H* and wildtype mice and analyzed using western blot with anti-ADAR1 monoclonal antibody. ADAR1 P110 isoform is dominantly expressed in brain tissue. As an IFN-stimulated protein, the P150 isoform of ADAR1 was detected in the *Adar D1113H* mouse brains due to IFN-signaling pathway activation. ADAR1 protein levels in ADAR D1113H brains were slightly higher than that of wildtype mice. **Figure S3.** ISG expression and distribution in the brain cortex regions of 8-month-old mice. Formalin fixed paraffin embedded (FFPE) cortex sections of 8-month-old mice were stained with hematoxylin and eosin (H&E) (left) or in situ hybridization (ISH) for ISG-10 (middle) or CXCL15 (right). Cortical sections show no evidence of inflammatory infiltrate on H&E stain in WT or mutant mice. ISH in mutant mice shows strong labeling of cortical neurons for ISG-15 (middle) and microglia for CXCL10 (right) in mutant but not WT mice. Bar = 100 microns. **Figure S4.** ISG expression and distribution in the brain deep gray matter areas of 8-month-old mice. Formalin fixed paraffin embedded (FFPE) sections of 8-month-old mice were stained with hematoxylin and eosin (H&E) (left) or in situ hybridization (ISH) for ISG-15 (middle) or CXCL10 (right). Deep gray matter sections (V = lateral ventricle, CP = choroid plexus) showed no evidence of inflammatory infiltrate on H&E stain in WT or mutant mice. ISH in mutant mice showed strong labeling of cortical neurons and ependyma for ISG-15 (middle) and microglia and ependyma for CXCL10 (right) in mutant but not WT mice. Bar = 100 microns. **Figure S5.** IBA1 and GFAP staining of the *Adar*^*D1113H /D1113H*^*;ifih1*^*−/−*^ double-mutant mice. FFPE sections of 8-week-old mice *Adar*^*D1113H /D1113H*^*;ifih1*^*−/−*^ double-mutant mouse brains were stained with immunohistochemistry (IHC) for IBA1 (left) or GFAP (right). **A** On cortical sections (CTX), IHC in double-mutant and WT mice show similar labeling of microglia with IBA1 (left) and astrocytes with GFAP (right). **B** Deep gray matter sections (V = lateral ventricle, CP = choroid plexus) show similar labeling of microglia with IBA1 (left) and astrocytes with GFAP (right). Bar = 100 microns.

## Data Availability

All data generated or analyzed during this study are included in this published article.

## Abbreviations

AGS: Aicardi–Goutières syndrome
ADAR1: Adenosine deaminase acting on RNA 1
IFN: Interferon
ISG: Interferon-stimulated gene
MDA-5: Melanoma differentiation-associated protein 5
5-HTR2c: 5-Hydroxytryptamine receptor 2C
GRIA: Glutamate ionotropic receptor AMPA type subunit
CXCL: C-X-C motif chemokine ligand
IFIT-1: Interferon-induced protein with tetratricopeptide repeats 1
FFPE: Formalin fixed paraffin embedded
ISH: In situ hybridization
IHC: Immunohistochemistry
