# Supplementary material for: An AGS-associated mutation in ADAR1 catalytic domain results in early-onset and MDA5-dependent encephalopathy with IFN pathway activation in the brain
Source: J Neuroinflammation. 2022 Dec 1;19:285. doi: 10.1186/s12974-022-02646-0 (PMC9714073; doi:10.1186/s12974-022-02646-0)
Supplement: Supplementary file 1 — Additional file 1: Figure S1. PCR genotyping analysis of Adar D1113H mutant mice. The PCR genotyping condition was optimized to distinguish WT alleles from mutant alleles. Primers used for this genotyping methods yielded two bands of different sizes as indicated. Figure S2. Adar D1113H mutant mice expressed normal sized ADAR1 protein in brains. Brain protein extracts were prepared from Adar D1113H and wildtype mice and analyzed using western blot with anti-ADAR1 monoclonal antibody. ADAR1 P110 isoform is dominantly expressed in brain tissue. As an IFN-stimulated protein, the P150 isoform of ADAR1 was detected in the Adar D1113H mouse brains due to IFN-signaling pathway activation. ADAR1 protein levels in ADAR D1113H brains were slightly higher than that of wildtype mice. Figure S3. ISG expression and distribution in the brain cortex regions of 8-month-old mice. Formalin fixed paraffin embedded (FFPE) cortex sections of 8-month-old mice were stained with hematoxylin and eosin (H&E) (left) or in situ hybridization (ISH) for ISG-10 (middle) or CXCL15 (right). Cortical sections show no evidence of inflammatory infiltrate on H&E stain in WT or mutant mice. ISH in mutant mice shows strong labeling of cortical neurons for ISG-15 (middle) and microglia for CXCL10 (right) in mutant but not WT mice. Bar = 100 microns. Figure S4. ISG expression and distribution in the brain deep gray matter areas of 8-month-old mice. Formalin fixed paraffin embedded (FFPE) sections of 8-month-old mice were stained with hematoxylin and eosin (H&E) (left) or in situ hybridization (ISH) for ISG-15 (middle) or CXCL10 (right). Deep gray matter sections (V = lateral ventricle, CP = choroid plexus) showed no evidence of inflammatory infiltrate on H&E stain in WT or mutant mice. ISH in mutant mice showed strong labeling of cortical neurons and ependyma for ISG-15 (middle) and microglia and ependyma for CXCL10 (right) in mutant but not WT mice. Bar = 100 microns. Figure S5. IBA1 and GFAP staining of th [file 12974_2022_2646_MOESM1_ESM.pptx]

## Slide 1
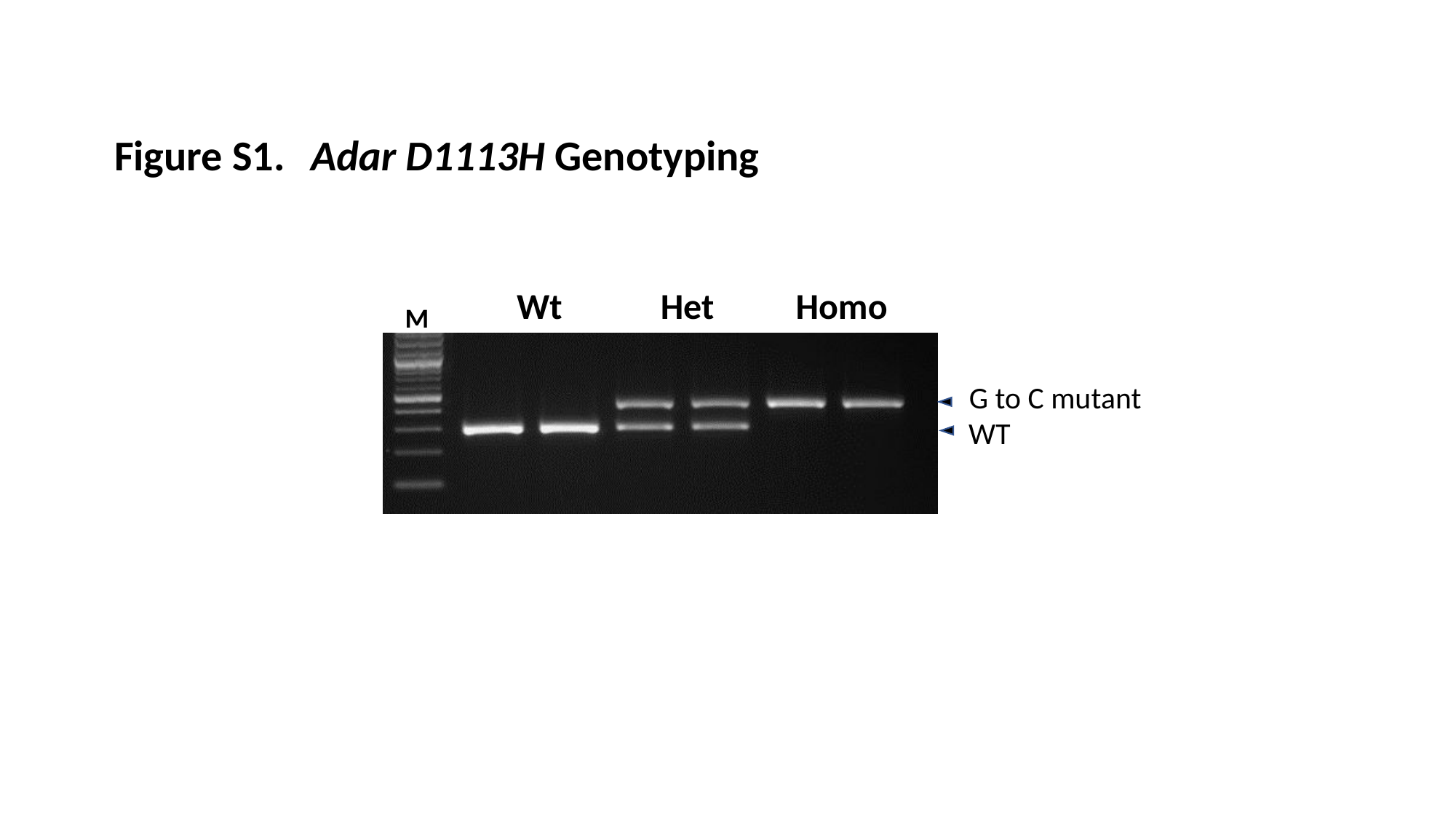

Figure S1.
Adar D1113H Genotyping
Wt Het Homo
M
G to C mutant
WT

## Slide 2
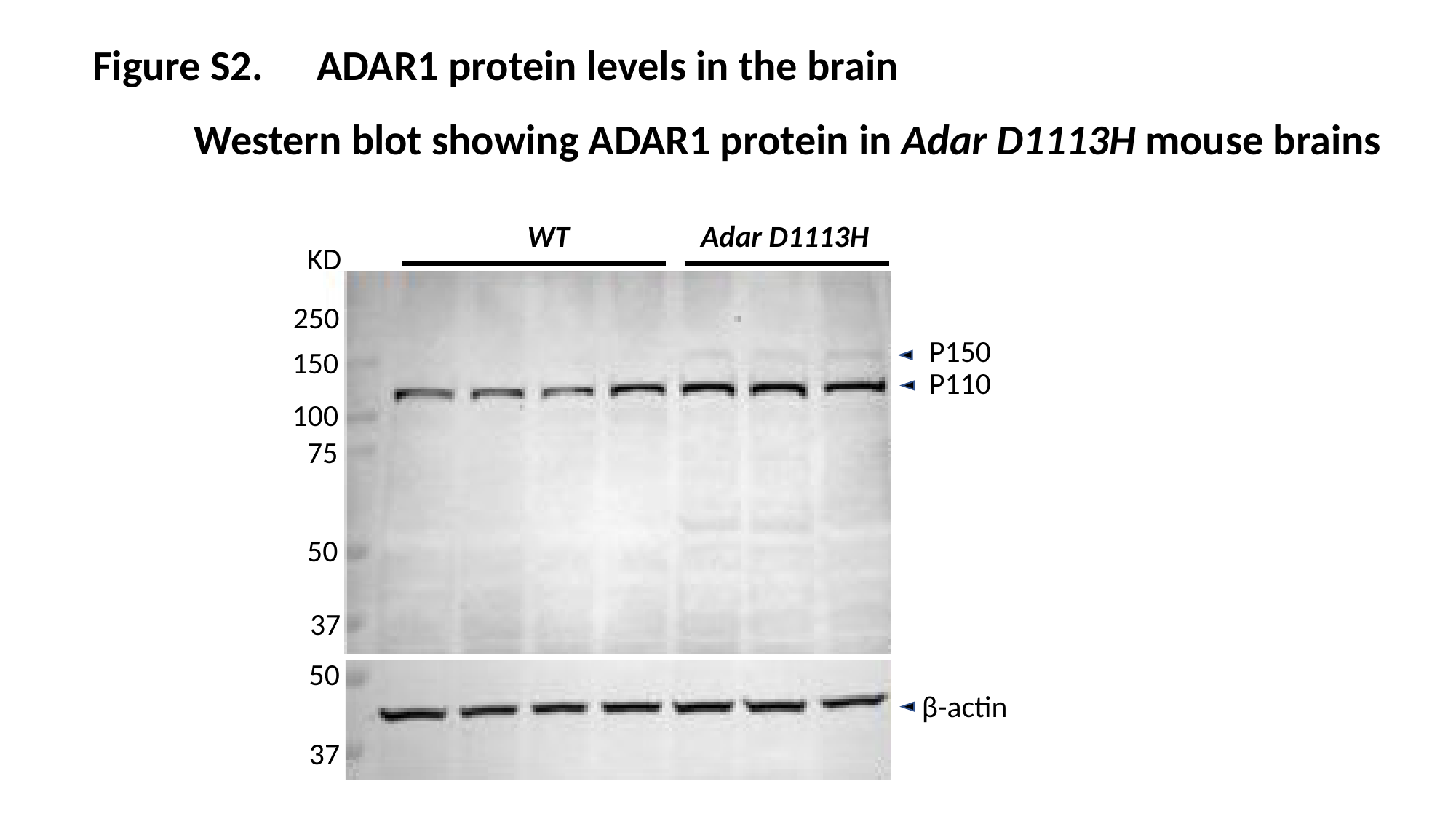

Figure S2.
ADAR1 protein levels in the brain
Western blot showing ADAR1 protein in Adar D1113H mouse brains
WT Adar D1113H
KD
250
P150
150
P110
100
75
50
37
50
β-actin
37

## Slide 3
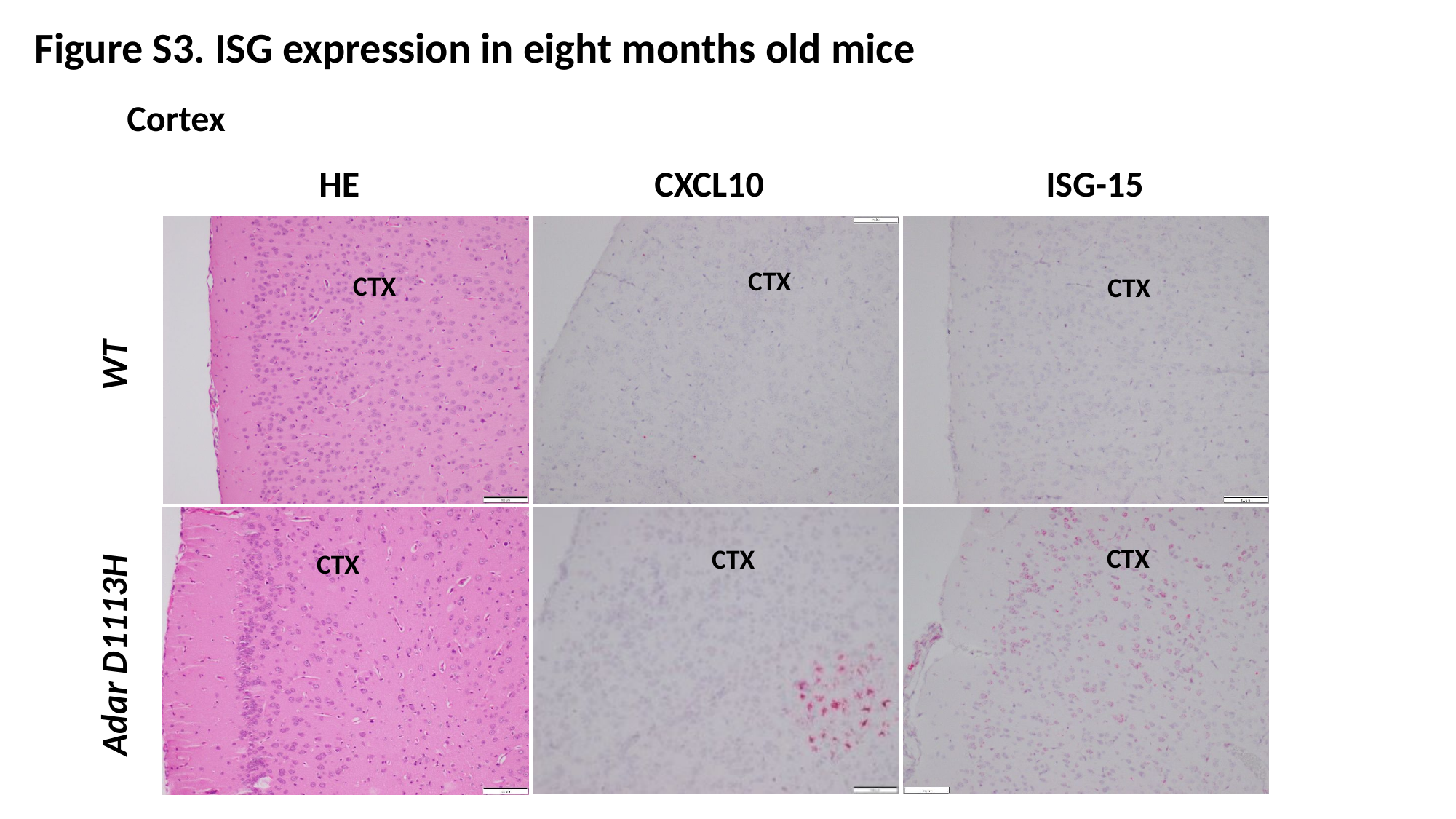

Figure S3. ISG expression in eight months old mice
Cortex
ISG-15
CXCL10
HE
WT
Adar D1113H
CTX
CTX
CTX
CTX
CTX
CTX

## Slide 4
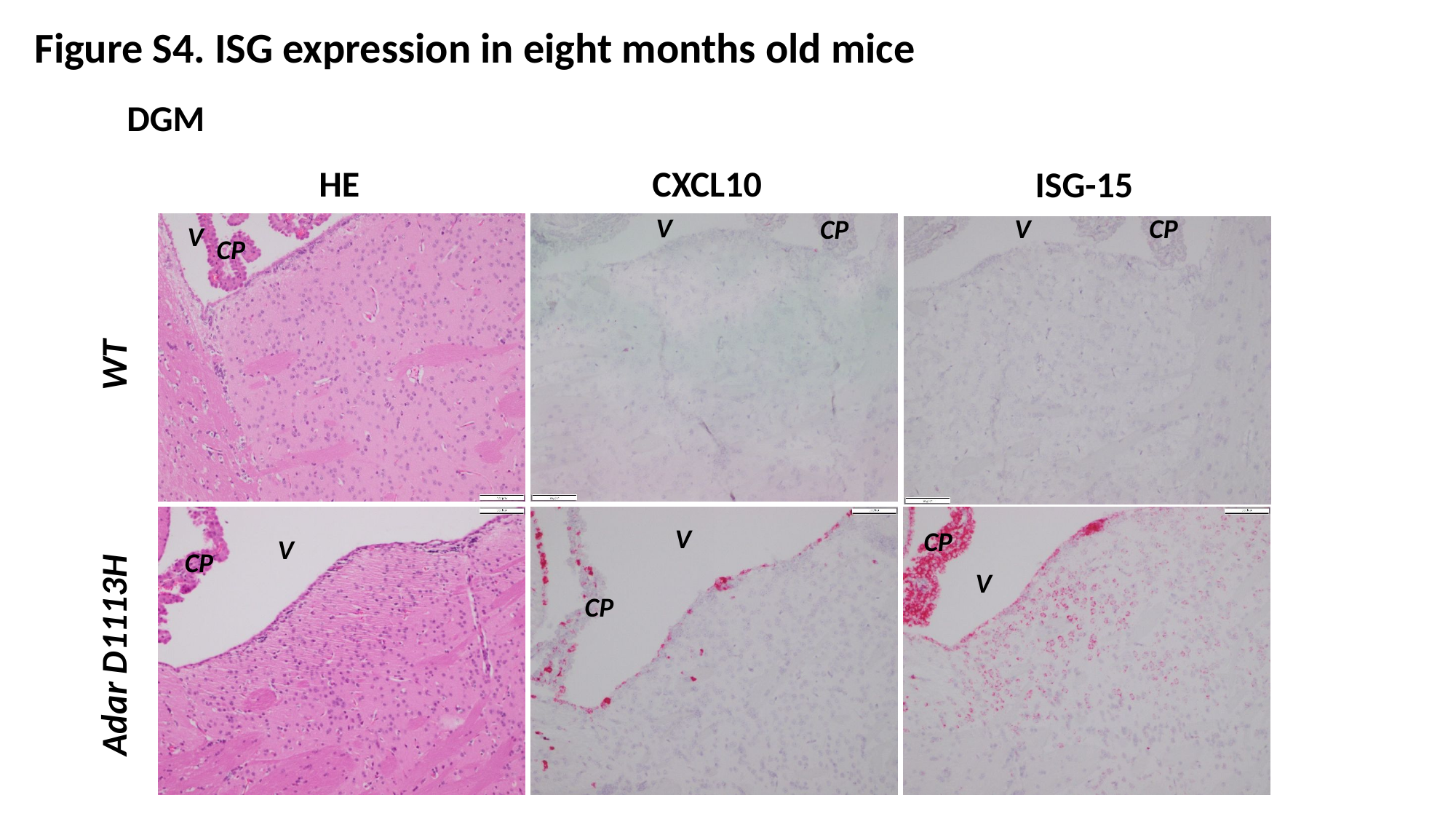

Figure S4. ISG expression in eight months old mice
DGM
CXCL10
HE
ISG-15
WT
Adar D1113H
V
V
CP
CP
V
CP
V
CP
V
CP
V
CP

## Slide 5
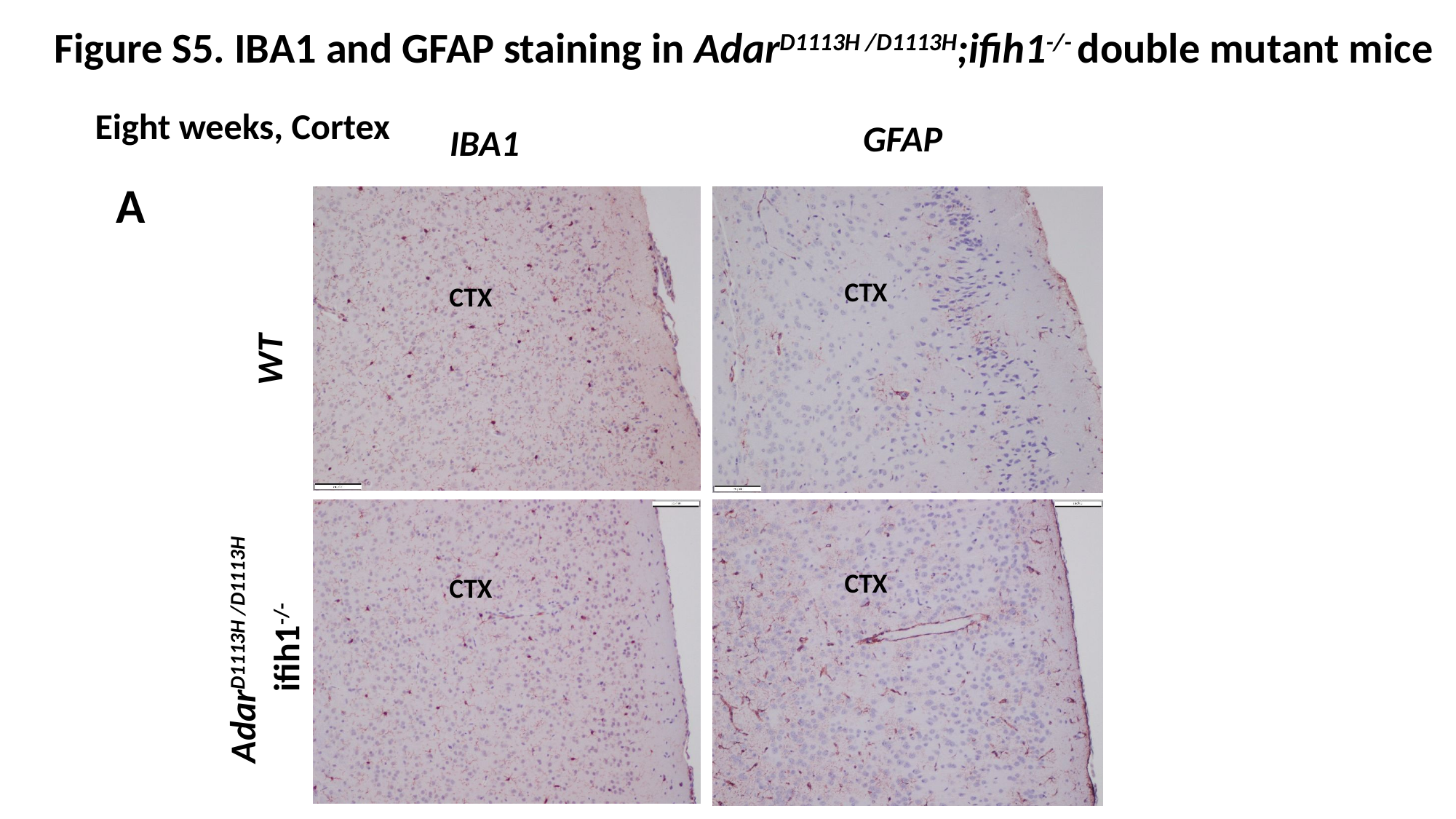

Figure S5. IBA1 and GFAP staining in AdarD1113H /D1113H;ifih1-/- double mutant mice
Eight weeks, Cortex
GFAP
IBA1
WT
A
CTX
CTX
CTX
CTX
AdarD1113H /D1113H
ifih1-/-

## Slide 6
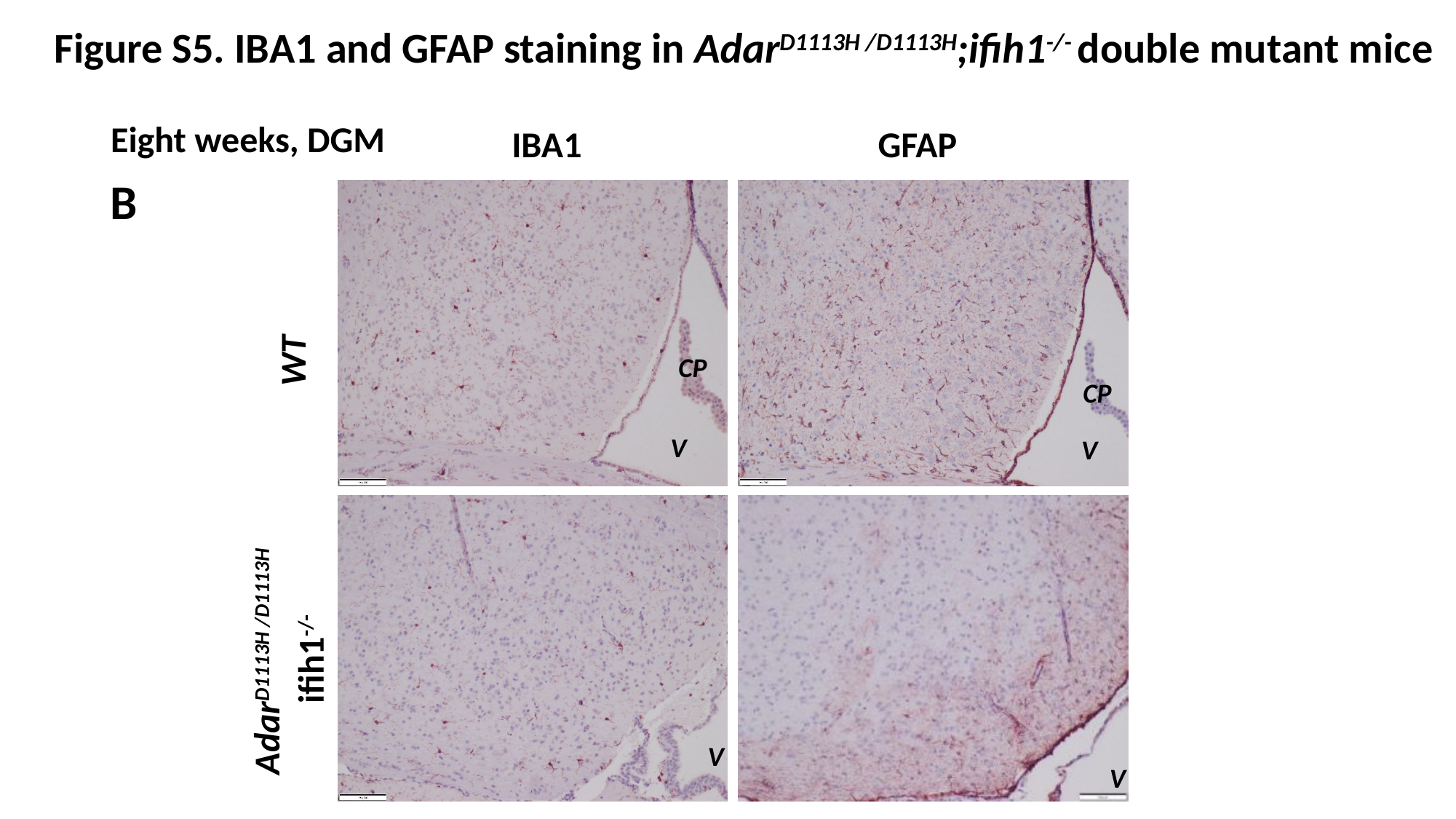

Figure S5. IBA1 and GFAP staining in AdarD1113H /D1113H;ifih1-/- double mutant mice
Eight weeks, DGM
IBA1
GFAP
WT
B
CP
CP
V
V
AdarD1113H /D1113H
ifih1-/-
V
V
